# Supplementary material for: Farnesoid X receptor activation by bile acids suppresses lipid peroxidation and ferroptosis
Source: Nat Commun. 2023 Oct 30;14:6908. doi: 10.1038/s41467-023-42702-8 (PMC10616197; doi:10.1038/s41467-023-42702-8)
Supplement: Supplementary file 1 — Supplementary Information [file 41467_2023_42702_MOESM1_ESM.pdf]

Supplementary Figures for

Farnesoid X Receptor activation by bile acids suppresses lipid peroxidation and ferroptosis

Juliane Tschuck[1], Lea Theilacker[1], Ina Rothenaigner[1], Stefanie A. I. Weiß[1], Banu Akdogan[2], Van Thanh Lam[3], Constanze Müller[4], Roman Graf[1], Stefanie Brandner[1], Christian Pütz[1], Tamara Rieder[5], Philippe Schmitt-Kopplin[4], Michelle Vincendeau[3], Hans Zischka[2,5], Kenji Schorpp[1], and Kamyar Hadian[1,§]

[1] Research Unit Signaling and Translation, Helmholtz Zentrum München, Neuherberg, Germany

[2] Institute of Molecular Toxicology and Pharmacology, Helmholtz Zentrum München, Neuherberg, Germany

[3] Institute of Virology, Helmholtz Zentrum München, Neuherberg, Germany

[4] Research Unit Analytical BioGeoChemistry, Helmholtz Zentrum München, Neuherberg, Germany

[5] Institute of Toxicology and Environmental Hygiene, Technical University Munich, School of Medicine, Munich, Germany

[§] Corresponding author: Kamyar Hadian, kamyar.hadian@helmholtz-muenchen.de

Supplementary Figure 1

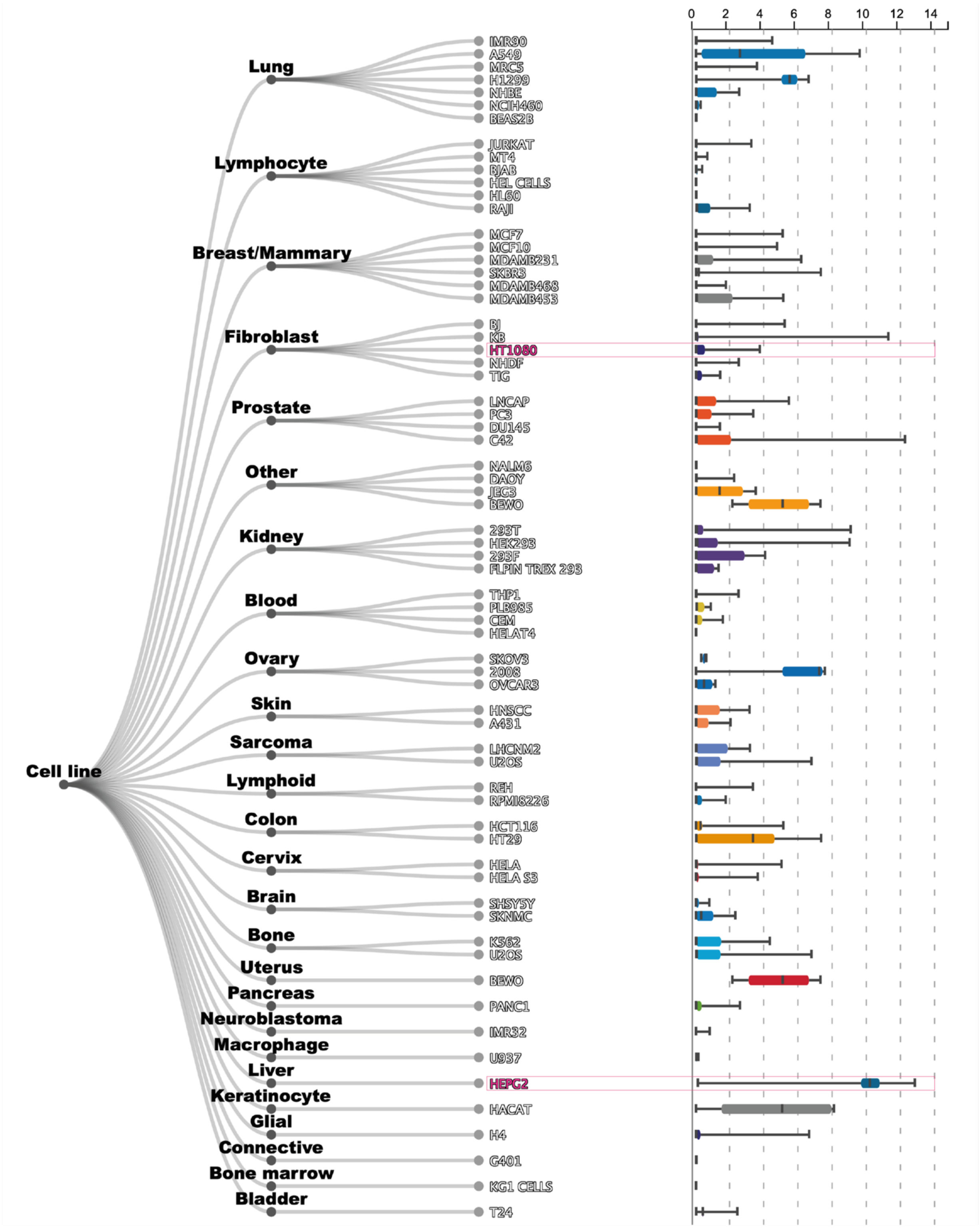

Supplementary Figure 1. Expression levels of FXR (NR1H4) across several cell lines extracted from ARCH[4] database (*Lachmann, A., Torre, D., Keenan, A.B., Jagodnik, K.M., Lee, H.J., Wang, L., et al., 2018. Massive mining of publicly available RNA-seq data from human and mouse. Nat Commun 9(1):1366.*)

Supplementary Figure 2

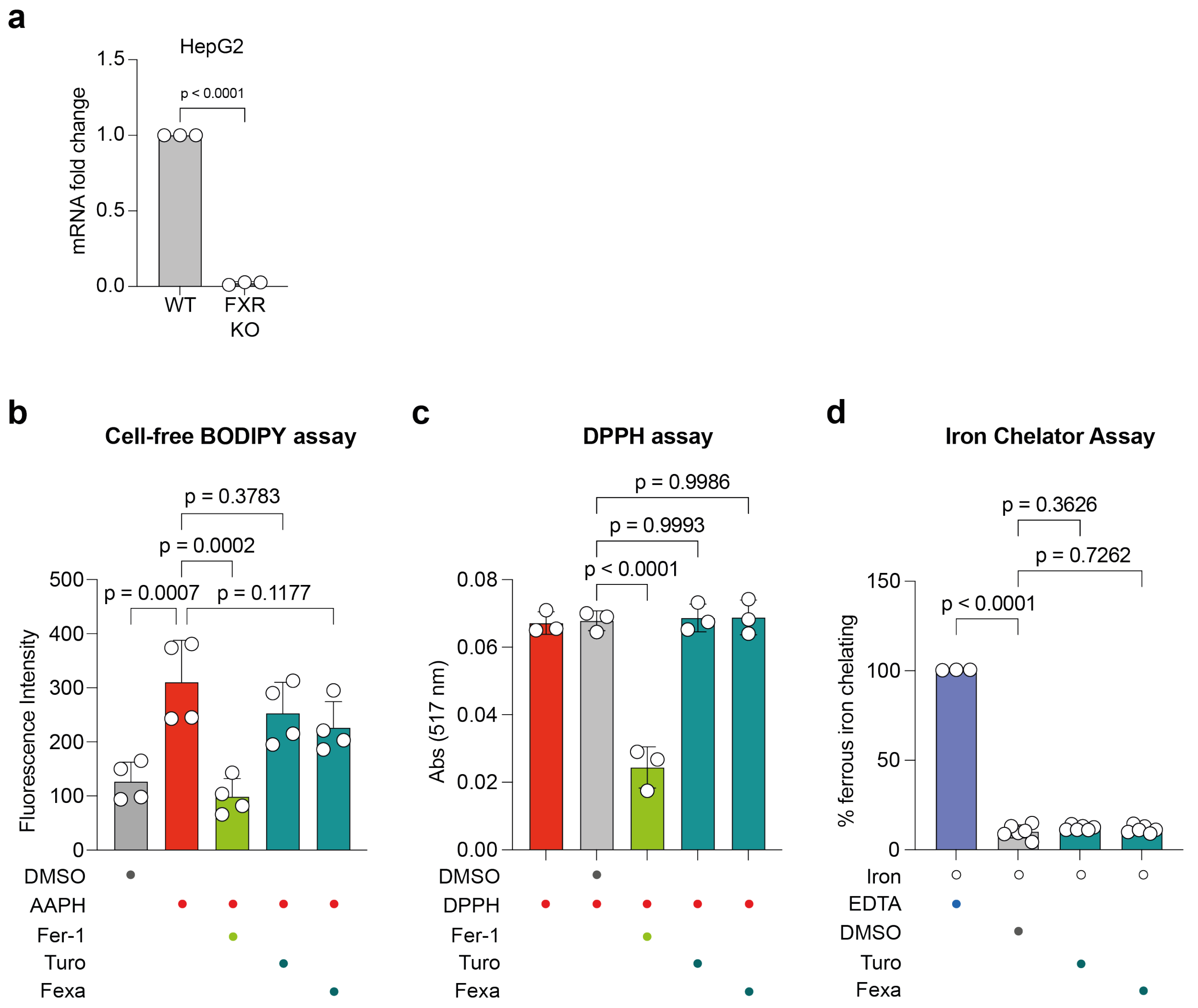

Supplementary Figure 2. **a** mRNA expression of FXR in wildtype (WT) and FXR knockout (KO) cells. Data are mean ± SD of *n* = 3 biological replicates; unpaired two-tailed *t*-test. **b** Turofexorate and Fexaramine have no antioxidant effects in a cell-free oxidizable BODIPY-C11 assay treated with 7.5 mM free-radical-producing 2,2′-azobis(2-methyl-propanimidamide) dihydrochloride (AAPH). In contrast, we observed significant decrease in oxidative fluorescence by Fer-1. Data are mean ± SD of *n* = 4 biological replicates; one-way ANOVA with Dunnett's test. **c** DPPH assay showed no antioxidant activity of Turofexorate and Fexaramine. All compounds were tested at a final concentration of 50 µM. DPPH was used at 0.05 mM. Data are mean ± SD of *n* = 3 biological replicates, one-way ANOVA with Tukey's test. **d** 12 µM Turofexorate or Fexaramine are not able to chelate iron (Fe(II)) in a Ferrozine-based assay. 100 µM EDTA were used as a positive control. Data are mean ± SD of *n* = 3 (EDTA) or 6 (all other treatments) biological replicates; one-way ANOVA with Dunnett's test.

Supplementary Figure 3

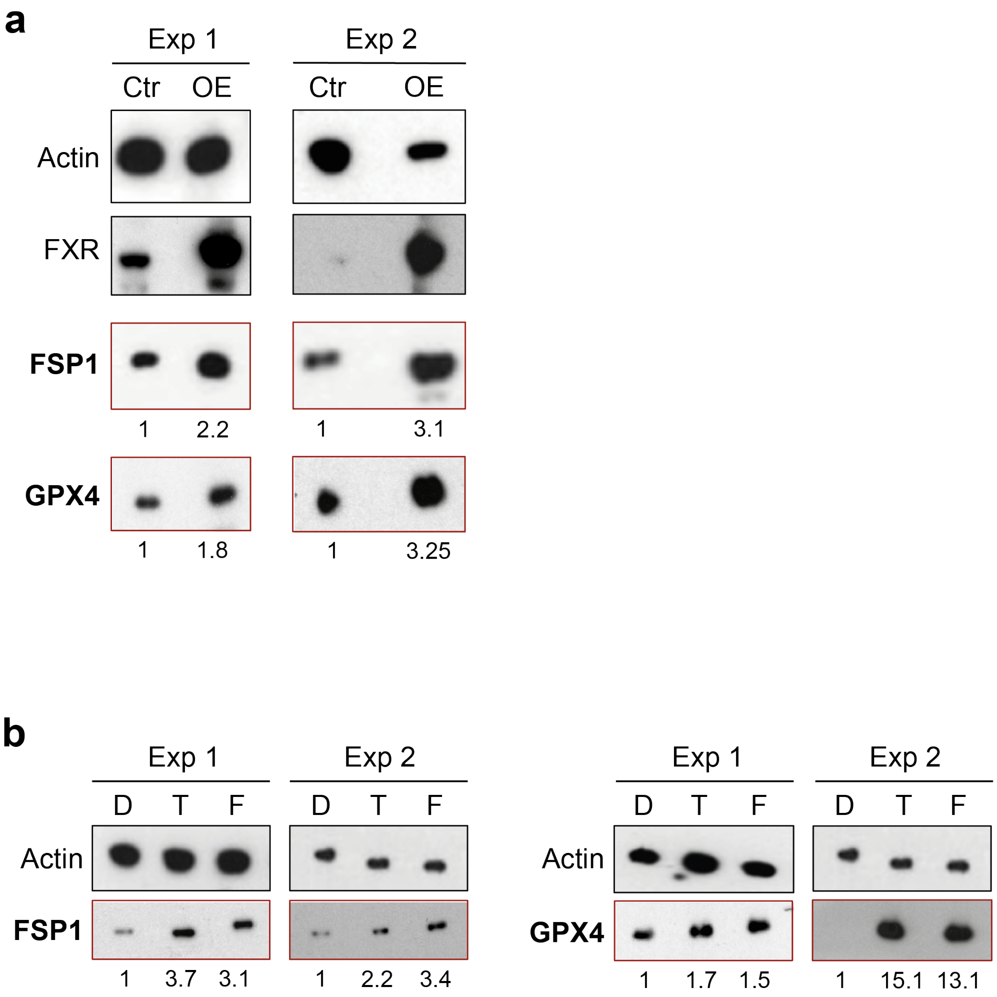

Supplementary Figure 3. **a** Western Blots of HT-1080 cells overexpressing FXR show an upregulation of target genes FSP1 and GPX4. **b** Western Blots of HepG2 cells treated with 12 μM Turofexorate or Fexaramine for 2h show an upregulation of target genes FSP1 and GPX4. Bands were quantified using ImageJ and signals were normalized to β-Actin signals. Data from 2 biological replicates are shown. Antibodies are listed in Supplementary Tables 1 and 2.

Supplementary Figure 4

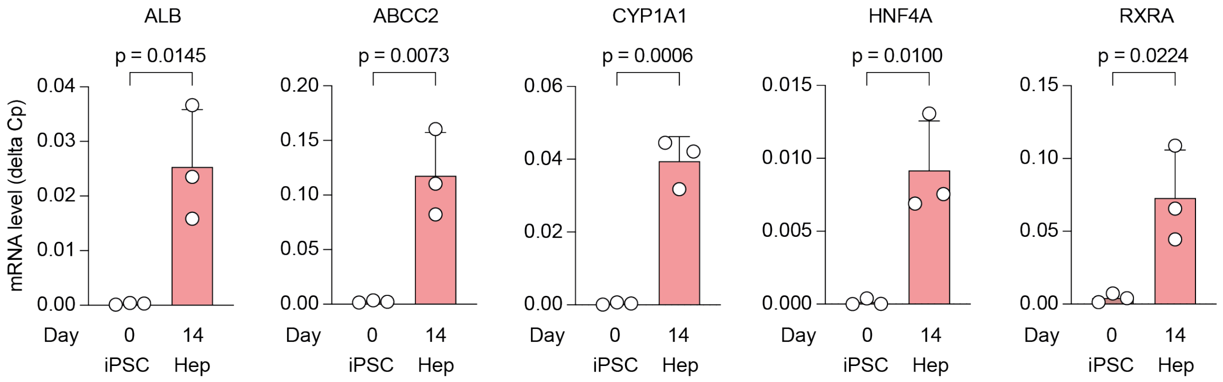

Supplementary Figure 4. Canonical differentiation markers of hepatocytes are upregulated on mRNA level after 14 days of differentiation compared to iPSCs at day 0. RP2 was used as a housekeeper gene for normalization. Data plotted are mean ± SD of *n* = 3 biological replicates; unpaired two-tailed t test.

Supplementary Figure 5

Figure 2a

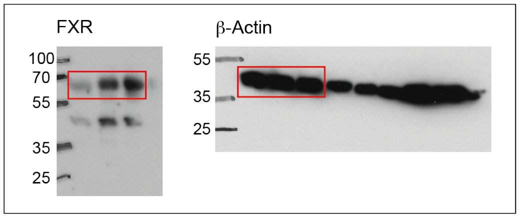

Figure 3b

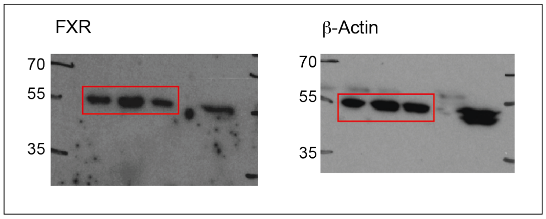

Supplementary Figure 3a

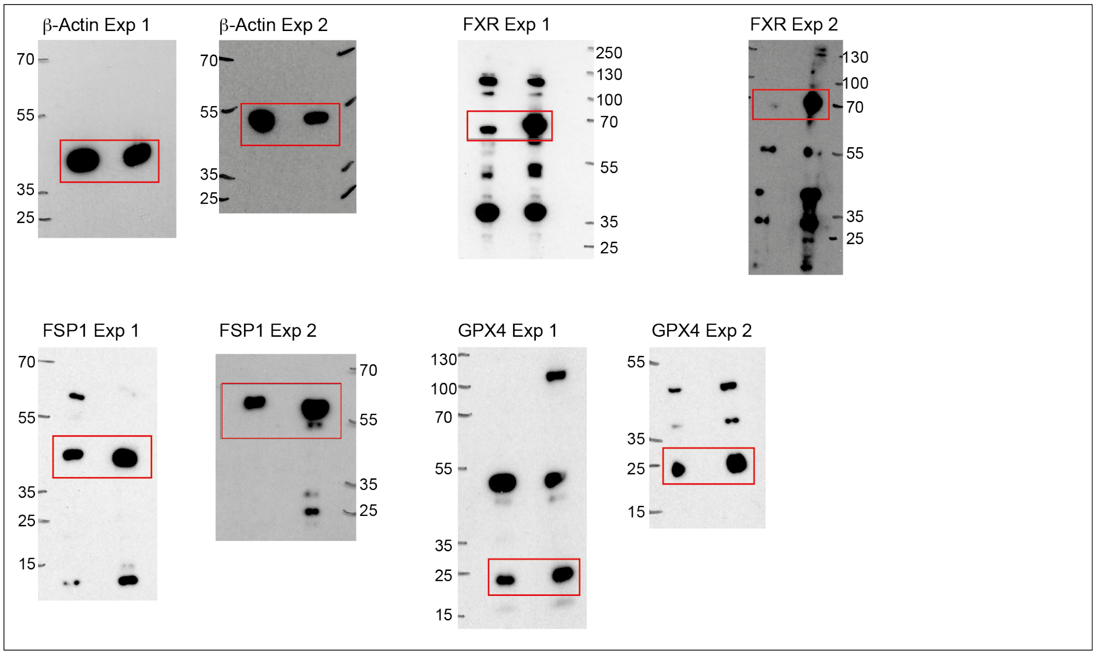

Supplementary Figure 3b

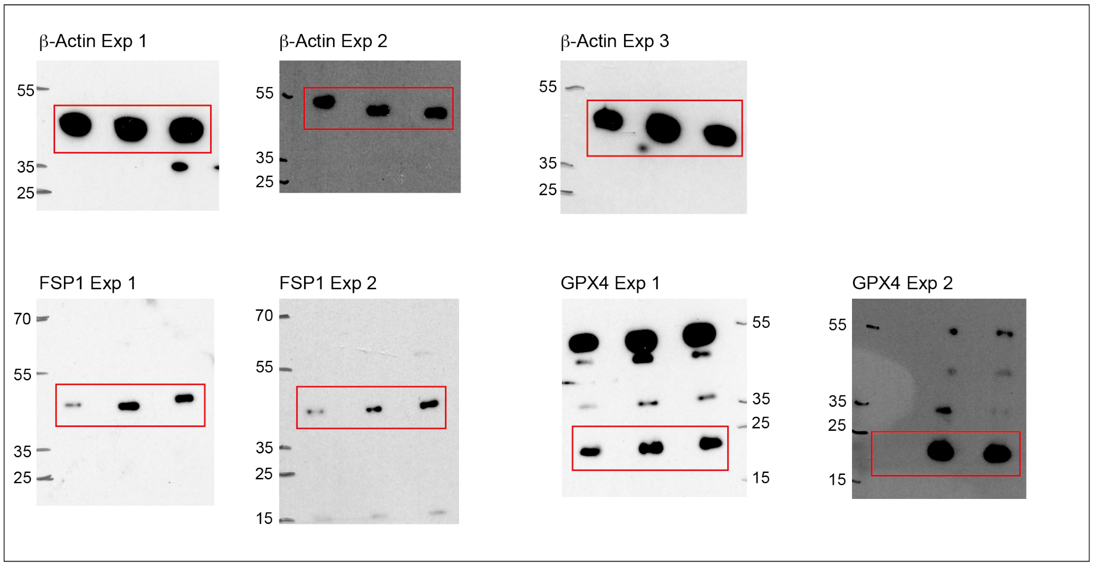

Supplementary Figure 5. Full scans of the Western Blots in Fig. 2a, 3b and Supplementary Fig. 3a, 3b

Supplementary Figure 6

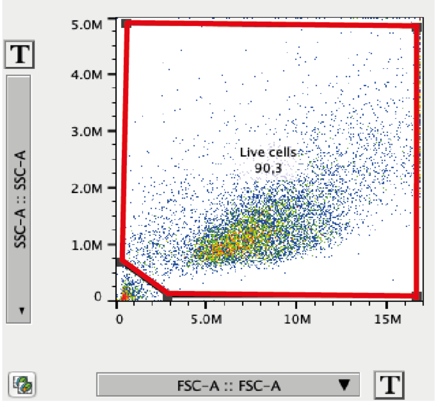

1. Exclude dead cells from scatter plot

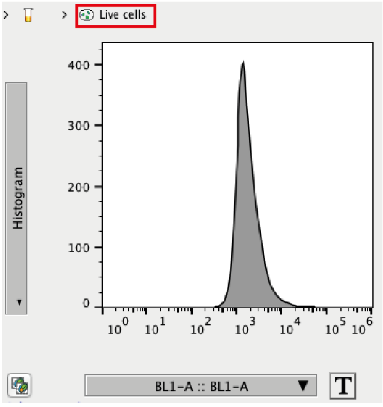

2. Plot histogram of live cells in BL1-A channel

	Sample Name	Subset Name	Count	Median : BL1-A
	Specimen Fexa 1.fcs	HT1080	9010	2588
	Specimen Turo 2.fcs	HT1080	3822	4957
	Specimen Fer-1 1.fcs	HT1080	9062	1441
	Specimen RSL3 2.fcs	HT1080	5374	16428
	Specimen DMSO.fcs	HT1080	9098	1585

3. Extract median intensities of BL1-A channel

Supplementary Figure 6. Flow cytometry gating strategy and analysis for Fig. 1f, 4a, 4c, and 4d.

Supplementary Tables

Supplementary Table 1:

**Primary Antibodies**	**Dilution**	**Species**	**Supplier**
b-Actin (C4)	1:500	mouse	sc-47778, Santa Cruz Biotechnology
NR1H4 (FXR)	1:500	rabbit	ab228949, Abcam
FSP1 (AMID)	1:1000	rabbit	PA5-103183, Thermo Fisher Scientific
GPX4	1:1000	rabbit	ab125066, Abcam

Supplementary Table 1:

**Secondary Antibodies**	**Dilution**	**Species**	**Supplier**
HRP-conjugated anti-mouse IgG	1:7500	donkey	715-035-150, Jackson ImmunoResearch Laboratories, Biozol
HRP-conjugated anti-rabbit IgG	1:7500	donkey	711-035-152, Jackson ImmunoResearch Laboratories, Biozol
