# Supplementary material for: Farnesoid X receptor activation by bile acids suppresses lipid peroxidation and ferroptosis
Source: Nat Commun. 2023 Oct 30;14:6908. doi: 10.1038/s41467-023-42702-8 (PMC10616197; doi:10.1038/s41467-023-42702-8)
Supplement: Supplementary file 3 — Description of Additional Supplementary Files [file 41467_2023_42702_MOESM3_ESM.pdf]

Description of Additional Supplementary Files

File name: Supplementary Data 1
Description: List of 3,684 compound IDs used in the compound screening in Figure 1a

File name: Supplementary Data 2
Description: Optimal compound concentrations used in apoptosis (Fig. 1c), necroptosis (Fig. 1d), FIN56 (Fig. 1e), and C11-BODIPY (Fig. 1f) testing.

File name: Supplementary Data 3
Description: Human and mouse qRT-PCR primers.
