# Supplementary material for: Farnesoid X receptor activation by bile acids suppresses lipid peroxidation and ferroptosis
Source: Nat Commun. 2023 Oct 30;14:6908. doi: 10.1038/s41467-023-42702-8 (PMC10616197; doi:10.1038/s41467-023-42702-8)
Supplement: Supplementary file 7 — Reporting Summary [file 41467_2023_42702_MOESM7_ESM.pdf]

nature portfolio

Corresponding author(s): Kamyar Hadian

Last updated by author(s): Nov 1, 2022

Reporting Summary

Nature Portfolio wishes to improve the reproducibility of the work that we publish. This form provides structure for consistency and transparency in reporting. For further information on Nature Portfolio policies, see our Editorial Policies and the Editorial Policy Checklist.

Statistics

For all statistical analyses, confirm that the following items are present in the figure legend, table legend, main text, or Methods section.

n/a	Confirmed	
☐	☒	The exact sample size (*n*) for each experimental group/condition, given as a discrete number and unit of measurement
☐	☒	A statement on whether measurements were taken from distinct samples or whether the same sample was measured repeatedly
☐	☒	The statistical test(s) used AND whether they are one- or two-sided<br>*Only common tests should be described solely by name; describe more complex techniques in the Methods section.*
☒	☐	A description of all covariates tested
☒	☐	A description of any assumptions or corrections, such as tests of normality and adjustment for multiple comparisons
☐	☒	A full description of the statistical parameters including central tendency (e.g. means) or other basic estimates (e.g. regression coefficient) AND variation (e.g. standard deviation) or associated estimates of uncertainty (e.g. confidence intervals)
☐	☒	For null hypothesis testing, the test statistic (e.g. *F*, *t*, *r*) with confidence intervals, effect sizes, degrees of freedom and *P* value noted<br>*Give P values as exact values whenever suitable.*
☒	☐	For Bayesian analysis, information on the choice of priors and Markov chain Monte Carlo settings
☒	☐	For hierarchical and complex designs, identification of the appropriate level for tests and full reporting of outcomes
☒	☐	Estimates of effect sizes (e.g. Cohen's *d*, Pearson's *r*), indicating how they were calculated

Our web collection on statistics for biologists contains articles on many of the points above.

Software and code

Policy information about availability of computer code

Data collection	PerkinElmer EnVision 2104 Multilabel plate reader; PerkinElmer Operetta high-content imaging/screening system; Roche LightCycler 480; Applied Biosystems Attune acoustic flow cytometer; Bruker Daltonics maXis coupled to a Waters Corporation UHPLC Acquity
Data analysis	PerkinElmer Columbus Software version 2.9.1; BD Life Sciences FlowJo v10.8.1 Software; Genedata Expressionist 13.5; R version 4.1.1; GraphPad Prism version 9.4, ImageJ 1.53k

For manuscripts utilizing custom algorithms or software that are central to the research but not yet described in published literature, software must be made available to editors and reviewers. We strongly encourage code deposition in a community repository (e.g. GitHub). See the Nature Portfolio guidelines for submitting code & software for further information.

Data

Policy information about availability of data

All manuscripts must include a data availability statement. This statement should provide the following information, where applicable:

- Accession codes, unique identifiers, or web links for publicly available datasets
- A description of any restrictions on data availability
- For clinical datasets or third party data, please ensure that the statement adheres to our policy

All data are available in the article and its Supplementary Information as well as in the online source file. Western Blot full scan images are shown in Supplementary Fig. 5. Compound library used in Fig. 1a is in Supplementary Table 1. Data in Supplementary Fig. 1 is available online from ARCH4 database.

March 2021

Human research participants

Policy information about studies involving human research participants and Sex and Gender in Research.

Reporting on sex and gender	No human research participants for this study
Population characteristics	No population data
Recruitment	n/a
Ethics oversight	n/a

Note that full information on the approval of the study protocol must also be provided in the manuscript.

Field-specific reporting

Please select the one below that is the best fit for your research. If you are not sure, read the appropriate sections before making your selection.

☒ Life sciences ☐ Behavioural & social sciences ☐ Ecological, evolutionary & environmental sciences

For a reference copy of the document with all sections, see nature.com/documents/nr-reporting-summary-flat.pdf

Life sciences study design

All studies must disclose on these points even when the disclosure is negative.

Sample size	No sample size calculations were performed. For biochemical or cell-based studies, at least three biological replicate experiments were tested that each consisted of several technical replicates. Exact numbers of replicates are stated in the figure legends.
Data exclusions	No data was excluded
Replication	Experiments were performed 3 times independently (exception: compound screening, ex vivo mouse experiment). Exact replicate numbers are stated in the figure legends.
Randomization	Cell line treatments were performed randomized because cells were seeded into plates with the same density across every well. Treatment method was the same during every experiment, e.g. well number 1 was always DMSO control etc. Isolated mouse hepatocytes from two mice were mixed before seeding cells for the different treatments. Mice were chosen randomly for experiments.
Blinding	Except for randomization, we did not actively take blinding measures in our experiments. However, we tried to avoid bias by performing experiments in several biological replicates and by not excluding important data.

Reporting for specific materials, systems and methods

We require information from authors about some types of materials, experimental systems and methods used in many studies. Here, indicate whether each material, system or method listed is relevant to your study. If you are not sure if a list item applies to your research, read the appropriate section before selecting a response.

Materials & experimental systems

n/a	Involved in the study	
☐	☒	Antibodies
☐	☒	Eukaryotic cell lines
☒	☐	Palaeontology and archaeology
☐	☒	Animals and other organisms
☒	☐	Clinical data
☒	☐	Dual use research of concern

Methods

n/a	Involved in the study	
☒	☐	ChIP-seq
☐	☒	Flow cytometry
☒	☐	MRI-based neuroimaging

Antibodies

Antibodies used	mouse anti-NR1H4/FXR antibody, ab228949 (Abcam); mouse anti-beta-Actin (C4), sc-47778 (Santa Cruz Biotechnology); rabbit anti-FSP1/AMID, PA5-103183 (Thermo Fisher Scientific); rabbit anti-GPX4 antibody, ab125066 (Abcam)<br>HRP-conjugated AffiniPure donkey anti-mouse IgG, 715-035-150 (Jackson ImmunoResearch); HRP-conjugated AffiniPure donkey anti-

	rabbit IgG, 711-035-152 (Jackson ImmunoResearch)<br>anti-4-Hydroxynonenal antibody (4-HNE), ab46545 (Abcam); anti-rabbit Alexa 488 antibody, A32731 (Thermo Fisher Scientific)

Validation

Only commercial antibodies were used, and these were rigorously validated by their distributors for specificity and functionality.

Validation statement for Western Blot antibodies:
Abcam: "Antibodies are validated in western blot using lysates from cells or tissues that we have identified to express the protein of interest. Once we have determined the right lysates to use, western blots are run and the band size is checked for the expected molecular weight. We will always run several controls in the same western blot experiment, including positive lysate and negative lysate. When possible, we also include knock-out (KO) cell lines as a true negative control for our western blots. We are always increasing the number of KO-validated antibodies we provide. In addition, we run old stock alongside our new stock. If we know the old stock works well, this also acts as a suitable positive control. If the western blot result gives a clear clean band and we are happy with the result from the control lanes, these antibodies will be passed and added to the catalog."

Statement Thermo Fisher: "Invitrogen antibodies are currently undergoing a rigorous two-part testing approach:
Part 1—Target specificity verification

This helps ensure the antibody will bind to the correct target. Our antibodies are being tested using at least one of the following methods to ensure proper functionality in researcher's experiments. Click on each testing method below for detailed testing strategies, workflow examples, and data figure legends.

Knockout—expression testing using CRISPR-Cas9 cell models
Knockdown—expression testing using RNAi to knockdown gene of interest
Independent antibody verification (IAV)—measurement of target expression is performed using two differentially raised antibodies recognizing the same protein target
Cell treatment—detecting downstream events following cell treatment
Relative expression—using naturally occurring variable expression to confirm specificity
Neutralization—functional blocking of protein activity by antibody binding
Peptide array—using arrays to test reactivity against known protein modifications
SNAP-ChIP™—using SNAP-ChIP to test reactivity against known protein modifications
Immunoprecipitation-Mass Spectrometry (IP-MS)—testing using immunoprecipitation followed by mass spectrometry to identify antibody targets

Part 2—Functional application validation

These tests help ensure the antibody works in a particular application(s) of interest, which may include
(but are not limited to):

Western blotting
Flow cytometry
ChIP
Immunofluorescence imaging
Immunohistochemistry

Most antibodies were developed with specific applications in mind. Testing that an antibody generates acceptable results in a specific application is the second part of confirming antibody performance."

Santa Cruz Biotechnology: "Santa Cruz Biotechnology is expanding our monoclonal antibody product line. We offer monoclonal antibodies directed against a broad range of mammalian and non-mammalian protein targets, representing essentially all targets covered by polyclonal antibodies. Primary antibodies directed to mammalian target proteins have been characterized for reactivity against mouse, rat and human proteins. Many of our mammalian antibodies are reactive with equine, bovine, canine, feline, caprine, porcine and ovine protein targets and are suitable for veterinary research. Primary antibodies directed to non-mammalian target proteins, including proteins of bacterial, viral, plant, zebrafish and Drosphila origin, are available. Our antibodies are recommended for use in most assays including Western blot, immunoprecipitation, immunostaining, and flow cytometry. We also offer a wide variety of secondary antibodies, control immunoglobulin and control sera for a large selection of species."

Eukaryotic cell lines

Policy information about cell lines and Sex and Gender in Research

Cell line source(s)	HT-1080 and HepG2 were purchased from ATCC; MEFs were a gift from Daniel Krappmann (Helmholtz Munich)
Authentication	Cell lines were not further authenticated
Mycoplasma contamination	Cell lines were regularly tested for mycoplasma, and were always negative for mycoplasma
Commonly misidentified lines<br>(See ICLAC register)	No commonly misidentified lines were used

Animals and other research organisms

Policy information about studies involving animals; ARRIVE guidelines recommended for reporting animal research, and Sex and Gender in Research

Laboratory animals	41-week-old male C57BL/6 mice (WT and hetero) were used
Wild animals	No wild animals
Reporting on sex	Male C57BL/6 mice were used for ex vivo mouse study. Sex-based analysis was not relevant for this study.
Field-collected samples	No field-collected samples
Ethics oversight	All animal experiments were conducted under the ethical guidelines for the care and use of laboratory animals of the Helmholtz Munich

Note that full information on the approval of the study protocol must also be provided in the manuscript.

Flow Cytometry

Plots

Confirm that:

☒ The axis labels state the marker and fluorochrome used (e.g. CD4-FITC).

☒ The axis scales are clearly visible. Include numbers along axes only for bottom left plot of group (a 'group' is an analysis of identical markers).

☒ All plots are contour plots with outliers or pseudocolor plots.

☒ A numerical value for number of cells or percentage (with statistics) is provided.

Methodology

Sample preparation	HT-1080 and HepG2 were used in flow cytometry studies; after treatment cells were harvested with 0.05% Trypsin-EDTA (Thermo Fisher) before 2 wash steps were conducted with PBS by spinning down the cells at 500 x g, 4°C for 5 min. Cells were stained with C11-BODIPY (Thermo Fisher Scientific) or with anti-4HNE antibody (ab46545 Abcam) and anti-rabbit Alexa 488 antibody (A32731, Thermo Fisher Scientific). For flow cytometry measurement, the cells were resuspended in 300 µl PBS and 10,000 events per condition were analyzed in the BL-1 channel.
Instrument	Applied Biosystems Attune acoustic flow cytometer
Software	BD Life Sciences FlowJo v10.8.1 Software
Cell population abundance	No sorting was performed. Flow cytometry was used for detection of lipid peroxidation upon C11-BODIPY and 4-HNE staining
Gating strategy	FSC/SSC gates were set to define workable cell population (live cells). Remaining population was used to generate histograms of the BL-1 channel for the different treatments. Median intensities of histograms of three replicate experiments were determined and plotted as bar graphs to evaluate statistics.

☒ Tick this box to confirm that a figure exemplifying the gating strategy is provided in the Supplementary Information.
